# Environmental Microbiome Characteristics and Disinfection Strategy Optimization in Intensive Dairy Farms: Bactericidal Efficacy of Glutaraldehyde-Based Combination Disinfectants and Regulation of Gut Microbiota

**DOI:** 10.3390/vetsci12080707

**Published:** 2025-07-28

**Authors:** Tianchen Wang, Tao He, Mengqi Chai, Liyan Zhang, Xiangshu Han, Song Jiang

**Affiliations:** College of Animal Sciences, Shihezi University, Shihezi 832000, China; 18083933115@163.com (T.W.); ht960704@163.com (T.H.); cmqtgzy@163.com (M.C.); 15729916090@163.com (L.Z.); ybwrsys@163.com (X.H.)

**Keywords:** bovine mastitis, quaternary ammonium compound (QAC) disinfectants, disinfection efficacy, environmental microbiota

## Abstract

As the primary biological risk threatening safe dairy production, bovine mastitis control highly relies on environmental disinfection measures. However, the mechanisms by which chemical disinfectants influence host–environment microbial interactions remain unclear. This study systematically investigated the disinfection efficacy and regulatory effects on microbial community composition and diversity of glutaraldehyde-benzalkonium chloride (BAC) and glutaraldehyde-didecyl dimethyl ammonium bromide (DAB) at recommended concentrations (2–5%), using 80 environmental samples from intensive dairy farms in Xinjiang, China. Combining 16S rDNA sequencing with culturomics, the results showed that BAC achieved a disinfection rate of 99.33%, higher than DAB’s 97.87%, and reduced the environment–gut microbiota similarity index by 23.7% via a cationic bacteriostatic film effect. Microbiome analysis revealed that BAC selectively suppressed Fusobacteriota abundance (15.67% reduction) and promoted Bifidobacterium proliferation (7.42% increase), enhancing intestinal mucosal barrier function through butyrate metabolism. In contrast, DAB induced Actinobacteria enrichment in the environment (44.71%), inhibiting pathogen colonization via bioantagonism. BAC’s long-acting bacteriostatic properties significantly reduced disinfection costs and mastitis incidence. This study first elucidated the mechanism by which quaternary ammonium compound (QAC) disinfectants regulate host health through “environment-gut” microbial interactions, providing a critical theoretical basis for developing precision disinfection protocols integrating “cost reduction-efficiency enhancement-risk mitigation.”

## Simple Summary

Bovine mastitis, a major threat to dairy production, necessitates effective environmental disinfection. This study evaluated the bactericidal efficacy of glutaraldehyde-based disinfectants (benzalkonium chloride, BAC, and didecyl dimethyl ammonium bromide, DAB) in intensive dairy farms and their effects on gut microbiota. The results showed BAC achieved a 99.33% disinfection rate, higher than DAB’s 97.87%. BAC reduced environment–gut microbiota similarity by 23.7%, suppressed Fusobacteriota, and promoted Bifidobacterium, enhancing intestinal health via butyrate metabolism. DAB enriched Actinobacteria in the environment, inhibiting pathogens through bioantagonism. BAC’s long-acting properties cut disinfection costs and mastitis incidence. This study reveals how quaternary ammonium compound disinfectants regulate host health via the “environment-gut” microbial axis, providing a basis for precision disinfection strategies to improve dairy sustainability and reduce economic losses.

## 1. Introduction

Milk quality and safety constitute the core monitoring aspect of the dairy production chain. The evaluation system includes key indicators such as total bacterial count (TBC) [1], somatic cell count (SCC) [2], antibiotic residue [3], and organoleptic properties (odor and appearance) [4]. Among these, bovine mastitis, primarily induced by environmental pathogenic microorganisms, has emerged as the foremost biological risk factor constraining safe milk production. This disease not only triggers inflammatory responses in mammary tissue, leading to alterations in milk composition and reduced milk yield (losses can reach 30–70% of the production from individual affected quarters), but also causes immune system stress, predisposing to secondary infections and significantly elevating culling rates in dairy herds [5]. According to the International Dairy Federation (IDF), the global annual direct economic loss attributed to bovine mastitis exceeds USD 35 billion [6]. Its economic burden now surpasses that of reproductive disorders, ranking as the most costly dairy cattle disease.

Current strategies for controlling bovine mastitis face significant limitations. Vaccines are constrained by the serotype diversity of pathogenic bacteria and may lack cost effectiveness in commercial dairy operations. Antibiotic therapy is prone to promoting the spread of resistant bacteria and carries risks of drug residue. Traditional management practices often fail to interrupt the persistent environmental pathogen transmission cycle [7].

Research indicates that most clinical mastitis cases are directly associated with pathogen colonization in environmental reservoirs such as milking equipment and bedding materials [8]. This makes Cleaning and Disinfection (C&D) a critical control point for breaking infection sources. Effective C&D protocols function through a dual mechanism: physical removal of organic soil eliminates microbial attachment substrates, while chemical disinfectants inactivate residual pathogens, thereby reducing the total viable count (TVC) on teat skin contact surfaces by 2–3 log_10_. However, disinfection efficacy is constrained by multiple factors, including disinfectant–pathogen match, the degree of organic matter interference, and adherence to standard operating procedures (SOPs) [9]. For instance, oxidizing disinfectants are readily inactivated by organic matter, rendering them unsuitable for heavily contaminated floors or livestock housing with organic debris and biofilms [10]. While phenolic compounds maintain efficacy better than iodophors and quaternary ammonium compounds (QACs) in the face of organic load, their use is restricted in many regions due to toxicity concerns for humans and animals [11]. Consequently, QAC–glutaraldehyde combination formulations have become the mainstream choice for modern farm disinfection, owing to their broad-spectrum antimicrobial activity and relatively environmentally friendly profile [12].

Existing research predominantly focuses on the immediate bactericidal effect of chemical disinfectants, yet lacks systematic assessment of how disinfectants impact environmental microbial community structure and their subsequent interactions with the animal gut microbiota. Traditional methods like plate counting only reflect the immediate kill rate of culturable microorganisms and cannot resolve the structural alterations disinfectants impose on microecosystems. Advances in microbiome research have confirmed complex microbial interactions between teat skin and the barn environment—opportunistic pathogens in bedding can colonize the teat surface via contact transmission, while excessive disinfection-induced environmental microbial dysbiosis can thus disrupt dairy cattle gut microbiota homeostasis via pathways like drinking water and feed [13,14]. Such microecological disturbances may precipitate multiple risks: on one hand, disinfectant residues can compromise the intestinal mucus barrier, reducing the abundance of beneficial bacteria like lactobacilli [15]; on the other hand, sublethal concentrations of disinfectants that exert the selective pressure may accelerate the horizontal gene transfer (HGT) of antimicrobial resistance genes (ARGs) within the environment. Therefore, establishing precision disinfection strategies based on microbial community modulation has become a crucial scientific imperative for safeguarding animal health and dairy product safety.

This study innovatively combines 16S rDNA high-throughput sequencing and culturomics to systematically evaluate the bactericidal efficacy of two glutaraldehyde-based combination disinfectants at recommended concentration gradients (2–5%) against environmental microbiota in dairy barns. By analyzing shifts in microbial community structure, it elucidates the impact of these disinfectants on the bovine intestinal microbiome. The findings will provide a theoretical foundation for developing precision disinfection protocols grounded in microecological balance, offering significant practical value for containing resistant pathogen spread, ensuring safe milk production, and mitigating the economic burden of dairy cattle diseases.

## 2. Materials and Methods

### 2.1. Dairy Barns

This study was conducted between July and November 2024. Two freestall barns were selected from a demonstration farm operated by a large dairy company in Tacheng Prefecture, Xinjiang, China. Prior to disinfection, farm workers cleared organic debris and excreta from the barns, followed by high-pressure washing. Farm workers documented disinfection protocols, including disinfectant names and application concentrations. Both barns housed lactating Holstein cows in a freestall system. Disinfection procedures were randomly assigned to two groups (see Table 1).

### 2.2. Sample Collection and Processing

A total of 360 bedding samples were collected from the two barns (180 per barn). Bedding disinfection efficacy was chosen as the indicator for evaluating overall barn disinfection effectiveness due to its close proximity to cows. Fresh bedding material was aseptically sampled using sterile spatulas from three depths: surface layer, middle layer (3 cm depth), and deep layer (5 cm depth). The samples were immediately placed into 3 mL of phosphate-buffered saline (PBS). The samples were transported to the laboratory within 2 h for microbiological analysis. The samples were serially diluted in sterile saline and plated onto Nutrient Agar. The plates were incubated aerobically at 37 °C for 18 h for enumeration of viable aerobic mesophilic bacteria.

Additionally, 80 environmental surface swab samples (40 per barn) were collected 7 days post-disinfection (to allow for expected microbial community recovery). Sampling targeted walls (including posts, windowsills, girders, feed rails, doors, and partitions), water troughs, and manure contact points. Samples of the same type were pooled for subsequent analysis. Sterile cotton-tipped swabs, moistened with sterile neutralizing buffer or saline, were used to vigorously swab defined surface areas (0.5–1 m^2^, area adjusted based on surface type). Each swab sample weighed approximately 1 g, Swabs were placed into sterile cryovials. Samples designated for sequencing analysis were immediately flash-frozen on dry ice and stored at −80 °C until processing.

### 2.3. Genomic DNA Extraction and Sample Analysis

Total genomic DNA was extracted from each sample using a magnetic bead-based nucleic acid extraction kit (TianGen Biochemical Technology Co., Ltd., Beijing, China), following the manufacturer’s protocol. DNA quality and concentration were assessed using 1% agarose gel electrophoresis and spectrophotometry (NanoDrop, Novogene Co., Ltd., Beijing, China). DNA was purified by 1% agarose gel electrophoresis as necessary. The hypervariable V3–V4 region of the bacterial 16S rRNA gene was amplified via polymerase chain reaction (PCR) using universal primers: 515F (5′-GTGYCAGCMGCCGCGGTAA-3′) and 806R (5′-GGACTACHVGGGTWTCTAAT-3′). PCR amplification conditions followed established protocols. PCR amplicons were purified using a DNA purification and recovery kit (TianGen Biochemical Technology Co., Ltd., Beijing, China). Library preparation was performed following standard Illumina protocols. Purified amplicon libraries were sequenced on an Illumina NovaSeq 6000 platform (Novogene Co., Ltd., Beijing, China) using a 2 × 250 bp paired-end configuration.

### 2.4. Bioinformatics Analysis

Raw sequencing reads were subjected to quality control and processing: Paired-end reads were merged using FLASH (v1.2.11). Merged reads underwent quality filtering (trimming low-quality bases, removing reads with ambiguous bases), primer removal, and chimera detection/removal to generate high-quality non-chimeric sequences (Effective Tags). Amplicon Sequence Variants (ASVs) were inferred from the high-quality sequences using the DADA2 pipeline within QIIME2 (v2022.2 or later), including error correction, denoising, and removal of residual chimeras. ASVs were taxonomically classified against a reference database (e.g., SILVA v138 or Greengenes 13_8) using a naive Bayes classifier trained on the specific primer set. Relative abundance histograms depicting the top 10 most abundant taxa at various taxonomic levels (Phylum, Class, Order, Family, Genus) were generated per sample using QIIME2.

Microbial alpha diversity within samples was assessed using the Shannon index (species diversity and evenness), Simpson index (dominance), and Chao1 index (richness estimator), calculated in QIIME2. Beta diversity (differences in microbial community composition between samples/groups) was analyzed using Principal Coordinates Analysis (PCoA) based on Bray–Curtis dissimilarity matrices or weighted/unweighted UniFrac distances. Calculations and visualizations were performed using R software (v4.0.3) with packages like phyloseq, vegan, and ggplot2. Linear Discriminant Analysis Effect Size (LEfSe) analysis (using LEfSe v1.1.01 or an equivalent plugin within QIIME2/Galaxy) was employed to identify differentially abundant taxa (potential biomarkers) across the three sampling depths or treatment groups. Statistically significant features were identified using the Kruskal–Wallis test (*p*-value < 0.05), followed by Linear Discriminant Analysis (LDA). Features with an LDA score > 3.5 were considered significant biomarkers.

## 3. Results

### 3.1. Analysis of Disinfection Efficacy for Two Disinfectants

**Figure 1:**
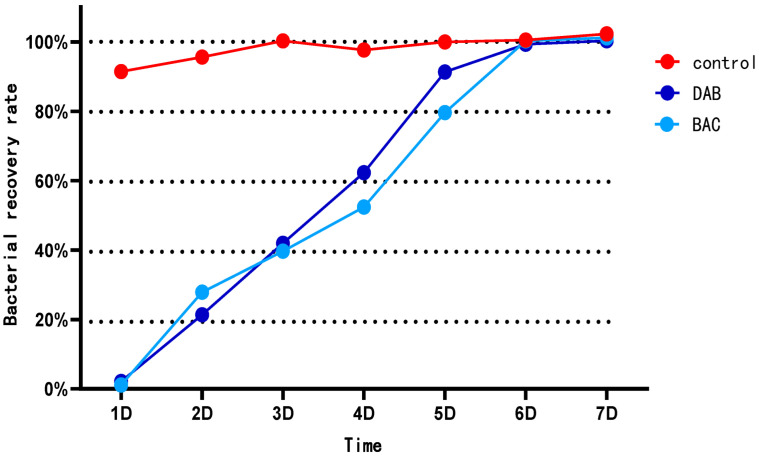
Spring environmental bacterial dynamics.

The experimental results demonstrated that the BAC group (benzalkonium chloride-based formulation) exhibited the highest disinfection efficacy, achieving a 99.33% reduction rate. The DAB group (didecyl dimethyl ammonium bromide-based formulation) showed the next highest efficacy, with a 97.87% reduction rate. Following disinfection, bacterial regrowth commenced on Day 2, with regrowth rates increasing to 39.67–45.31% by Day 3. The most rapid proliferation occurred on Day 5, reaching 52.39–62.34%. By Day 6, the total viable count (TVC) had progressively returned to pre-disinfection levels (Table 2, Figure 1).

### 3.2. 16S Amplicon Sequencing Analysis of Disinfectant Effects on Gut and Environmental Microbiota

#### 3.2.1. Impact of Disinfection Regimens on Microbial Community Alpha Diversity

**Figure 2:**
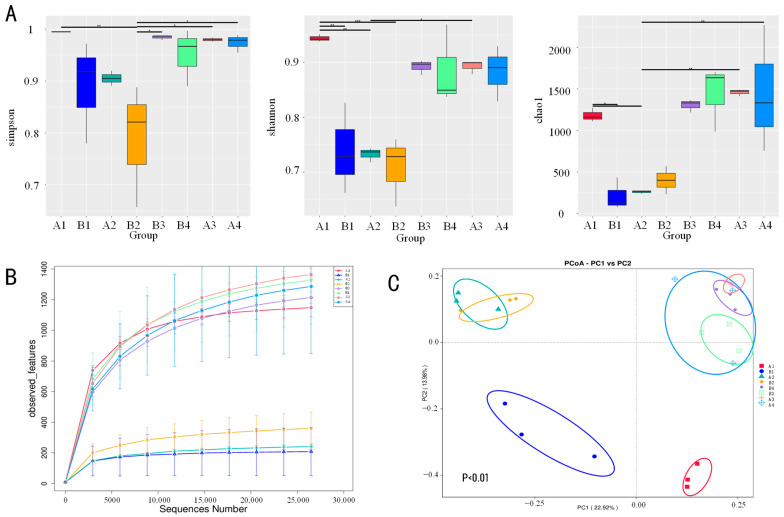
Changes in gut and environmental microbiota composition after exposure to DAB and BAC. (**A**) Comparison of Simpson, Shannon, and Chao1 indices for gut and environmental microbiota between DAB and BAC groups (n = 10 samples/group). (**B**) Rarefaction curves of OTUs clustered at 97% sequence similarity across groups (n = 10 samples/group). (**C**) Principal coordinates analysis (PCoA) based on Unweighted UniFrac distances for different samples. Note: A1, A2, A3, and A4 represent gut, drinking water, environmental, and teat surface samples from the DAB group, respectively. B1, B2, B3, and B4 represent corresponding samples from the BAC group. The same applies to the following figures. Asterisk denotes statistically significant differences * *p* < 0.05; ** *p* < 0.01; *** *p* < 0.001.

Alpha diversity, measured by species richness (Chao1 index) and diversity/evenness (Shannon index), was compared using Student’s *t*-tests. Fecal samples in the BAC group exhibited significantly lower Chao1 and Shannon indices than the DAB group (*p* < 0.05), while the Simpson index showed no significant difference. Similarly, drinking water, environmental, and teat skin samples from the BAC group had significantly lower Chao1 indices (*p* < 0.05), with no significant differences in Shannon or Simpson indices (Figure 2A). Rarefaction curves plateaued, indicating sufficient sequencing depth to capture microbial diversity and reliable data for subsequent analysis (Figure 2B).

#### 3.2.2. Microbial Community Beta Diversity and Structural Differentiation

Beta diversity was analyzed via Principal Coordinate Analysis (PCoA) based on weighted UniFrac distance matrices for fecal, drinking water, environmental, and teat skin samples. Fecal microbiota showed significant separation between DAB and BAC groups (*p* < 0.01), reflecting distinct disinfectant-induced gut microbial alterations. Drinking water samples formed unique clusters, significantly separated from other sample types (*p* < 0.01), highlighting their distinct structural composition. Environmental and teat skin samples also clustered significantly within groups (*p* < 0.01) (Figure 2A).

#### 3.2.3. Phylum- and Genus-Level Analysis of Microbial Community Composition

##### Dominant Phyla Distribution

**Figure 3:**
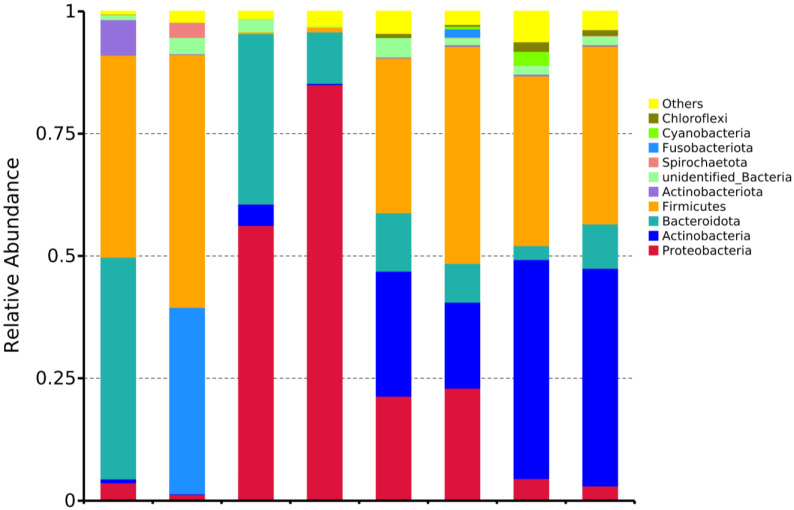
Relative abundance of major bacterial phyla in cow gut and environment after DAB and BAC exposure (bar plot) (n = 10 samples/group).

Phylum-level analysis showed (Figure 3, Table 3) that eight dominant phyla (relative abundance > 1%) were detected in both groups, sequentially Proteobacteria, Actinobacteria, Bacteroidota, Firmicutes, Spirochaetota, Fusobacteriota, Cyanobacteria, and Chloroflexi. Specifically:

Gut microbiota: The DAB group was dominated by Firmicutes (51.71%), while the BAC group was dominated by Bacteroidota (45.28%);

Drinking water: The dominant phylum in the DAB group was Proteobacteria (56.27%), whereas the BAC group was dominated by Firmicutes (44.38%);

Environmental samples: The DAB group was dominated by Actinobacteria (44.71%), while the BAC group showed an absolute dominance of Proteobacteria (85.06%);

Mammary surface: The dominant phylum in the DAB group was Actinobacteria (44.46%), whereas the BAC group was dominated by Firmicutes (31.64%).

##### Dominant Genera Distribution

**Figure 4:**
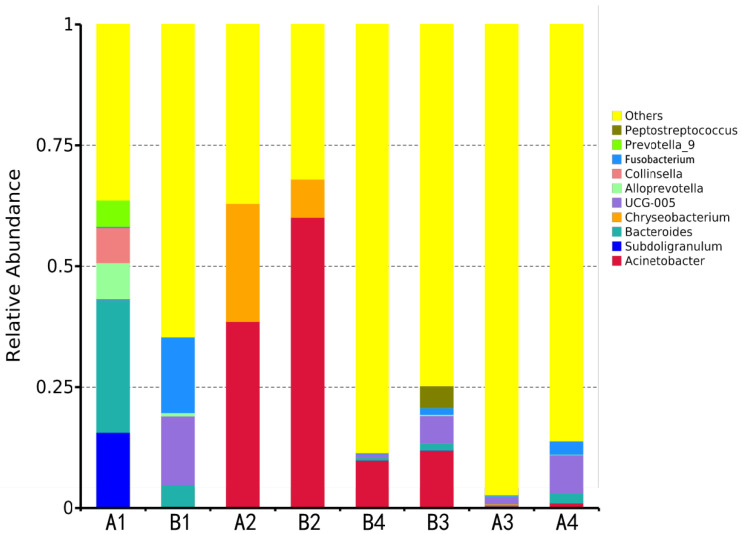
Relative abundance of major bacterial genera in cow gut and environment after DAB and BAC exposure (bar plot) (n = 10 samples/group).

Genus-level analysis showed (Figure 4, Table 4) that ten core genera (relative abundance > 1%) were identified in both groups, including Acinetobacter, Bacteroides, Chryseobacterium, Rikenellaceae RC9 gut group, Collinsella, etc. Key differences were observed as follows:

Gut microbiota: the DAB group was dominated by the Rikenellaceae RC9 gut group (15.63%), while the BAC group was dominated by Bacteroides (27.42%);

Drinking water: Acinetobacter was the common dominant genus in both groups, with significantly higher abundance in the BAC group than in the DAB group (60.14% vs. 38.60%);

Environmental and mammary samples: the dominant genera in the DAB group were UCG-005 (1.56%) and UCG-005 (7.86%), respectively, while the BAC group was dominated by Acinetobacter in both (11.95% and 9.88%).

#### 3.2.4. OTU Sharing Patterns and Community Similarity

**Figure 5:**
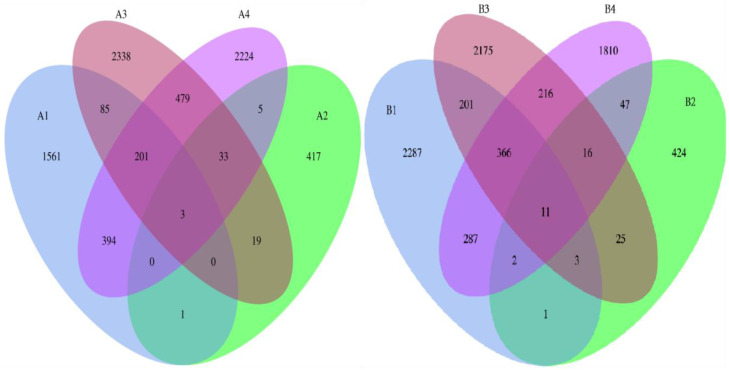
Venn diagram of shared OTUs across different groups in DAB and BAC treatments.

Venn diagram analysis (Figure 5) showed that the number of shared OTUs between gut samples and environmental/mammary samples in the DAB group was 1 and 394, accounting for 5.45% and 25.24% of the total gut OTUs, respectively. In the BAC group, the shared OTUs between gut samples and environmental/mammary samples were 201 and 287, accounting for 8.79% and 12.55%, respectively, indicating certain homology between mammary and gut microbiota. The Sørensen–Dice index further revealed (Table 5) that the similarity between gut and environmental microbiota was low in both groups (4.37% vs. 8.27%), while the similarity with mammary microbiota was higher (39.92% vs. 12.29%). Notably, gut microbiota shared no OTUs with drinking water microbiota, with a similarity of 0, reflecting the barrier effect on microbial transmission across different habitats.

#### 3.2.5. Biomarker Screening for Differentially Enriched Genera

**Figure 6:**
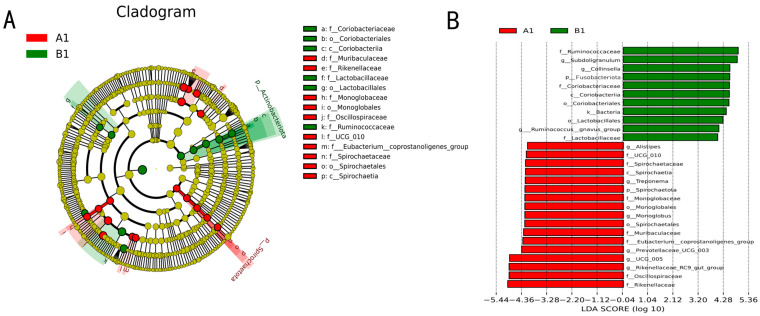
Microbial abundance differences between DAB and BAC groups (n = 10 samples/group). (**A**) Cladogram. (**B**) LDA (Linear Discriminant Analysis) distribution. Linear discriminant analysis effect size (LEfSe) was used to identify microbial abundance differences between the two groups. Note: The bar chart of LDA value distribution displays species with an LDA score greater than the set value (default setting is 4), i.e., biomarkers that are statistically different between groups.

LEfSe analysis (Figure 6A) showed that at the phylum level, Spirochaetota was significantly enriched in the DAB group (A1 sample), while Actinobacteria were specifically enriched in the BAC group (B1 sample). At the genus level, the DAB group’s gut microbiota was enriched with carbohydrate-metabolism-related genera such as Alistipes, Monoglobus, and Prevotellaceae UCG-003, whereas the BAC group was enriched with protein degradation-related genera including Subdoligranulum, Collinsella, and Ruminococcus gnavus group. These results suggest that different disinfection methods may affect host metabolic potential by altering the composition of gut functional genera.

## 4. Discussion

In recent years, intensive dairy farming has widely adopted chemical disinfectants to control pathogen transmission, but this practice faces dual challenges: overuse of commercial disinfectants is linked to environmental pollution [16] and zoonotic disease outbreaks [17]. While disinfectants provide immediate bactericidal effects, farm managers often overlook their potential adverse impacts on the gut microbiome, immune function, and public health risks [18]. Previous studies show disinfectant exposure—via residue ingestion or environment–host interactions—alters gut microbiota structure and induces immune dysregulation [19]. However, systematic research is lacking on the ecological effects of quaternary ammonium compound (QAC) disinfectants (e.g., benzalkonium chloride/BAC, didecyl dimethyl ammonium bromide/DAB) in dairy farms, particularly their mechanisms for disrupting the complex ruminant gut ecosystem [20], a knowledge gap urgently needing address given global dairy intensification [20].

Data from the International Dairy Federation (IDF) show increasing proportions of large-scale commercial dairy farms. While high-density housing boosts efficiency, it also increases disinfectant application intensity per unit area. In this context, disinfectant-induced changes in environmental microbiota (e.g., antimicrobial resistance gene (ARG) enrichment, functional microbiota depletion) may reshape the bovine gut microbiome via the “environment-feed-gut” transmission chain, impacting nutrient metabolism, immune homeostasis, and mammary health [21]. However, scientific understanding of disinfectant-mediated microbiome interactions across the environment–host interface and their quantitative effects on production performance remains insufficient, hindering optimization of safe and efficient disinfection protocols.

This study confirmed that the glutaraldehyde-benzalkonium chloride (BAC) formulation achieved a 99.33% reduction rate, demonstrating superior and longer-lasting bactericidal activity compared to glutaraldehyde-didecyl dimethyl ammonium bromide (DAB). BAC’s enhanced efficacy is attributed to its quaternary ammonium group’s stronger cationic surfactant properties, enabling competitive electrostatic binding to the lipopolysaccharide (LPS) layer of Gram-negative bacteria, which disrupts membrane integrity and enhances glutaraldehyde penetration [22,23]. In contrast, DAB’s hydrophobic alkyl chains intercalate into the peptidoglycan layer of Gram-positive bacteria, leading to selective inhibition of phyla like Firmicutes (e.g., Actinobacteriota) [24]. This explains the higher Actinobacteriota abundance in DAB environmental samples—Actinobacteria, as major producers of secondary metabolites (including antibiotics), may suppress opportunistic pathogens (e.g., *Escherichia coli*) via bioantagonism [25]. Additionally, BAC forms a long-lasting positively charged bacteriostatic film on surfaces, reducing species richness (23.7% Chao1 index decrease). DAB, with its larger molecular weight, undergoes micelle aggregation in organic-rich environments (e.g., fecal zones), reducing effective bacteriostatic concentration. This aligns with the lower environment–gut microbiome similarity index in the BAC group, collectively demonstrating BAC’s advantage in blocking cross-host pathogen transmission.

Environmental microbial differences indirectly influence gut microbiota via cow contact/ingestion. LEfSe analysis showed reduced Fusobacteriota abundance in BAC vs. DAB guts, possibly due to BAC’s quaternary ammonium group binding sulfated polysaccharides in intestinal mucus, locally elevating pH to 6.8–7.2 and inhibiting *Fusobacterium* spp. proliferation [26,27]. Concurrently, Parabacteroides’ abundance increased in BAC guts. Notably, Actinobacteriota enrichment in BAC-treated environments and guts may further modulate host immune homeostasis via bacterial secondary metabolites (e.g., short-chain fatty acids, SCFAs).

Previous studies established Firmicutes, Bacteroidota, Actinobacteriota, and Proteobacteria as the dominant phyla in the bovine intestinal tract [28]. Our findings align with this pattern in the DAB group, where Firmicutes (51.71%) and Bacteroidota (38.08%) predominated. However, the BAC group exhibited a distinct dominance pattern characterized by Bacteroidota, Firmicutes, and Actinobacteriota. Crucially, BAC induced significant enrichment of Actinobacteriota and suppression of Fusobacteriota compared to DAB, highlighting disinfectant-specific modulation. While studies by Joseph D. Sciurba et al. [29] and Dias M.F. et al. [30] reported negligible impacts of chemical disinfectants on murine gut alpha-diversity indices, the complex rumen fermentation system of ruminants may amplify the ecological consequences of disinfectant residues. The BAC-mediated suppression of Fusobacteriota is clinically relevant, as reduced abundance of this phylum correlates with decreased colitis incidence [31,32]. Supporting this, Swidsinski et al. [32] documented strong associations between Fusobacteriota species (particularly *Clostridium* spp.) and acute appendicitis. These collective findings provide a mechanistic rationale for BAC implementation in ruminant health management.

The gut microbiome exhibits functional plasticity, dynamically adjusting its community structure and metabolic output in response to environmental cues [33]. Substantial evidence confirms environmental influences on livestock gut microbiota, including dairy cattle [34], piglets [35], and poultry [36]. Our weighted UniFrac-based PCoA revealed significant beta-diversity separation across disinfectant regimens and sample types (gut, drinking water, environment, teat skin). High community similarity between gut and environmental samples indicates ecological connectivity, whereas water samples formed distinct clusters, suggesting inadequate disinfection protocols for water troughs [37]. Notably, Acinetobacter exceeded 60% relative abundance in all water samples, implicating inadequate trough sanitation. The reduced microbial similarity between gut and teat/environmental samples post-BAC disinfection suggests diminished microbial exchange, potentially lowering mastitis risk. This is critical given the documented cross-contamination pathways of fecal matter between bedding, teat surfaces, and farm facilities via animal movement and management practices [38,39].

Sørensen–Dice similarity analysis demonstrated significantly lower gut–environment microbiome overlap in the BAC group versus DAB (23.7% reduction), confirming that BAC’s cationic bacteriostatic film effectively impedes cross-host pathogen transmission. Although Acinetobacter persisted in water systems, BAC reduced its abundance in teat samples by 17.3%, indicating partial mitigation of waterborne risks through synergistic environmental disinfection and teat sanitation [40,41]. This supports implementing integrated disinfection strategies with targeted interventions for water systems [42,43] and rigorous pre-/post-milking teat hygiene.

Economically, BAC’s microbiome-modulating properties offer substantial loss mitigation potential for dairy operations. Its superior efficacy (99.33% reduction) and prolonged bacteriostatic activity (48–72 h) enable reduced application frequency and concentration. In organic-matter-rich environments, BAC maintains efficacy without reapplication, reducing disinfection frequency in intensive farms from 3 times per week to 2 and lowering labor and material costs. This targeted disinfection avoids excessive inhibition of functional taxa like Actinobacteriota, preserving their bioantagonistic effects, which can reduce the incidence of mastitis and lower veterinary expenses—though specific figures require long-term farm-scale tracking—disrupting the “over-disinfection → dysbiosis → antimicrobial dependency” cycle.

From a production efficiency perspective, BAC-driven suppression of Fusobacteriota and enrichment of Parabacteroides optimizes gut ecosystem homeostasis [44]. Elevated beneficial microbiota enhances the feed conversion ratio (FCR) [45], reducing concentrate feed requirements. Concurrently, reinforced intestinal barrier function and immune modulation lower the incidence risks of colitis and mastitis. The 17.3% reduction in teat Acinetobacter abundance correlates with decreased mastitis risk [46], preventing losses from milk quality downgrades (due to elevated SCC) and premature culling.

Furthermore, BAC’s disruption of the “environment-gut microbiome axis” (23.7% reduced similarity) curtails pathogen dissemination in high-density housing, averting epidemic-related economic cascades. This preventive strategy aligns with “low-cost–high-return” risk management principles, offering synergistic ecological and economic benefits for sustainable intensive dairy production.

## 5. Conclusions

This study, by comparing the bactericidal efficacy of two glutaraldehyde-based compound disinfectants and their regulatory effects on environmental microbiota in dairy farms, has confirmed, based on functional verification and 16S rDNA high-throughput sequencing, that glutaraldehyde-benzalkonium chloride (BAC) possesses the advantages of high-efficiency sterilization, long-lasting bacteriostasis, and precise microecological regulation in dairy farm disinfection. By reducing the abundance of Fusobacteria and enhancing the metabolic activity of Parabacteroides, BAC significantly improves intestinal health and immune function, and lowers the risk of key diseases such as mastitis. Its long-lasting bacteriostatic film property enables structural optimization of disinfection costs, reducing antibiotic dependence and feed waste. The research findings not only provide a safe and efficient disinfection scheme for intensive dairy farms, but also construct an economic value chain of “cost reduction—efficiency enhancement—risk control” from the perspective of microbiome regulation, which holds important practical significance for recovering economic losses in animal husbandry caused by disease attrition and resource waste. In the future, cost–benefit analysis across different breeding scales can be further conducted to promote the industrial application and precise popularization of this strategy.

## Figures and Tables

**Table 1:** Disinfectant groups, number of barns treated, application parameters, and environmental controls.

Items	Didecyl Dimethyl Ammonium Bromide (DAB)	Benzalkonium Chloride (BAC)
Disinfectant Information	Manufacturer: Sichuan Dingjian Animal Health Co., Ltd., Sichuan, China Composition: 100 mL contains 10 g glutaraldehyde + 10 g didecyl dimethyl ammonium bromide	Manufacturer: Coventry Chemicals Ltd., Coventry, UK Composition: 100 mL contains 15 g glutaraldehyde + 10 g benzalkonium chloride
Barn Conditions	Independent barns housing 30 healthy lactating cows with rice straw bedding	Independent barns housing 30 healthy lactating cows with rice straw bedding
Sampling Time	7 days post-disinfection	7 days post-disinfection
Application Parameters	Equipment: electric sprayer (0.3 MPa) Application rate: 300 mL/m^2^	Equipment: electric sprayer (0.3 MPa) Application rate: 300 mL/m^2^
Environmental Controls	Temperature: 25 ± 2 °C Humidity: 50 ± 5% RH Ammonia concentration: 5.2–7.8 ppm Bedding pH: 6.5–7.2 Bedding dry matter content: oven-dried at 65 °C for 24 h, 35–40%	Temperature: 25 ± 2 °C Humidity: 50 ± 5% RH Ammonia concentration: 5.2–7.8 ppm Bedding pH: 6.5–7.2 Bedding dry matter content: oven-dried at 65 °C for 24 h, 35–40%

**Table 2:** Spring environmental bacterial dynamics.

Group	Before Disinfection (10^4^ CFU/mL)	After Disinfection (10^4^ CFU/mL)	Disinfection Rate (%)
Control group	82.00	75.00	8.54
DAB group	83.00	0.64	97.87
BAC group	76.00	0.51	99.33

**Table 3:** Dominant phyla composition in DAB and BAC group samples (relative abundance %).

Sample Type	DAB Group Dominant Phyla	BAC Group Dominant Phyla
Gut	*Firmicutes* (51.71%) *Bacteroidota* (38.08%)	*Bacteroidota* (45.28%) *Firmicutes* (41.30%) *Actinobacteria* (8.80%)
Drinking Water	*Proteobacteria* (56.27%) *Bacteroidota* (34.86%)	*Firmicutes* (44.38%) *Proteobacteria* (23.00%) *Actinobacteria* (17.57%) *Bacteroidota* (7.92%)
Environment	*Actinobacteria* (44.71%) *Firmicutes* (34.71%)	*Proteobacteria* (85.06%) *Bacteroidota* (10.53%)
Teat Surface	*Actinobacteria* (44.46%) *Firmicutes* (36.31%) *Bacteroidota* (9.11%)	*Firmicutes* (31.64%) *Actinobacteria* (25.55%) *Proteobacteria* (21.39%) *Bacteroidota* (11.95%)

**Table 4:** Dominant genera composition in DAB and BAC group samples (relative abundance %).

Sample Type	DAB Group Dominant Genera	BAC Group Dominant Genera
Gut	*Rikenellaceae_RC9_gut_group* (15.63%) *UCG-005* (14.23%) *Bacteroides* (4.83%)	*Bacteroides* (27.42%) *Fusobacterium* (15.67%) *Alloprevotella* (7.42%) *Collinsella* (7.27%) *Prevotella_9* (5.47%)
Drinking Water	*Acinetobacter* (38.60%) *Chryseobacterium* (24.43%)	*Acinetobacter* (60.14%) *Chryseobacterium* (7.93%)
Environment	*UCG-005* (1.56%) *Rikenellaceae_RC9_gut_group* (0.24%)	*Acinetobacter* (11.95%) *UCG-005* (5.65%)
Teat Surface	*UCG-005* (7.86%) *Rikenellaceae_RC9_gut_group* (2.74%) *Bacteroides* (2.11%)	*Acinetobacter* (9.88%) *UCG-005* (0.75%) *Rikenellaceae_RC9_gut_group* (0.17%)

**Table 5:** SIMPER analysis of bacterial community similarity (%).

Group	Drinking Water	Environment
Gut (DAB group)	0	4.37
Gut (BAC group)	0	8.27

## Data Availability

No new data were created or analyzed in this study. Data sharing is not applicable to this article.
